# Assisted Reproductive Technologies in Brazil: characterization of centers and profiles from patients treated

**DOI:** 10.5935/1518-0557.20200001

**Published:** 2020

**Authors:** Rosana Machin, Douglas Mendosa, Maria Helena Oliva Augusto, Pedro Augusto Araújo Monteleone

**Affiliations:** 1 Universidade de São Paulo, Faculdade de Medicina, Departamento de Medicina Preventiva, São Paulo, SP, Brazil; 2 Universidade Federal de São Paulo, Escola Paulista de Política, Economia e Negócios, Campus Osasco, Osasco, SP, Brazil; 3 Universidade de São Paulo, Faculdade de Filosofia, Letras e Ciências Humanas, Departamento de Sociologia, São Paulo, SP, Brazil; 4 Universidade de São Paulo, Faculdade de Medicina, Hospital das Clínicas, Centro de Reprodução Humana Mario Covas, São Paulo, SP, Brazil

**Keywords:** infertility, assisted reproductive technology, access

## Abstract

**Objective:**

The aim of this study was to analyze the application of assisted reproductive technologies (ART) in Brazil from the active clinics and the population served considering the changes in the last resolutions of the Federal Council of Medicine (CFM), which enabled the use of the techniques for anyone, regardless of their health insurance system, gender or marital status.

**Methods:**

This paper was based on the analysis from the “Reproductive Technologies and (in) fertility study: regulation, market and rights”. We used quantitative and qualitative methodologies. In this paper, we used the empirical data produced in the quantitative study. The quantitative online survey was carried out in 2016-2018, answered by 81 fertility clinics in Brazil about their performance in 2015-2016. We opted to use the REDCap Program, a web-based application for the construction and management of online surveys and databases. The questionnaire addressed the characteristics of services, practices performed, population served and existing forms of funding.

**Results:**

The questionnaires returned corresponded to 63.1% of the clinics in the southeast region. ART is mainly offered by 90.1% private clinics. We report that 63.8% of establishments have up to 20 employees; 44.5% have been in operation between 11 and 20 years. 85.1% of the clinics reported having treated non-Brazilian residents.

**Conclusions:**

There has been a significant increase in the provision of ART in Brazil. Access remains thoroughly dependent on its own financial resources. The new CFM resolutions have shifted from the prevailing concept of "health care" to "assisting with new family configurations”.

## INTRODUCTION

Assisted Reproductive Technologies (ART) have grown significantly since the first birth through in vitro fertilization (IVF) in 1978 in the United Kingdom. Health technologies applied to the reproductive field have been incorporated in an accelerated manner in diverse socio-cultural contexts. Reproductive technologies have emerged to treat infertility by assisting infertile couples to conceive, but these technologies have brought new possibilities for those who experience other forms of relationships, with same-sex partners or without partners, to consider the possibility of reproduction. With such expansion, the techniques were no longer focused on a health issues, and became available for other situations where reproduction was not possible.

In Brazil, the majority of ART services continue to be performed in the private sector as these services are not covered by the Brazilian Unified Health System (the SUS). There are, nevertheless, some services, most of which are linked to universities, that provide to those unable to afford a private clinic.

In 2005, the National Health Surveillance Agency (ANVISA) created the National Embryo Production System (SisEmbrio), a national compulsory registry of stem cells, tissues and banks, and an information system including records of the production of human embryos by IVF. ANVISA is a regulatory agency linked to the Ministry of Health. According to the agency’s data, in 2016, 141 services reported 33,790 cycles of assisted reproduction, with 66,597 cryopreserved embryos (65% of them coming from clinics located in southeast Brazil) (ANVISA, 2016).

The development of ARTs in Brazil was marked by a context of low regulation (Machin, 2018). There is no specific state law for these practices. To safeguard practices, Brazilian doctors follow ethical rules in the form of deontological norms relating to the techniques used in assisted reproduction. Since 1992, guidelines have been established by the Federal Council of Medicine (CFM) “for the use of assisted reproduction” (1957/10, 2013/13, 2121/15, 2168/17) (CFM, 2010; 2013; 2015; 2017).

In the 2121 resolution (2121/2015), the use of techniques on homosexual couples and single women is explicitly included. This wording appears to convey a context of apparent universality in the eligibility of access. However, since ARTs are not available within the scope of the public healthcare system, private clinics are the only option, and there remains a significant economic barrier.

The present study was developed to analyze the characteristics of active clinics in Brazil, the practices developed and the population served.

## MATERIALS AND METHODS

This paper was based on empirical material produced in the frame of a broader research focused on assisted reproduction services in Brazil in 2016-2018, and the main implications of their practices in the field of healthcare technologies resulting from regulatory changes. Due to the nature of the study, we chose both a quantitative and qualitative methodology. In this paper, we use the empirical data produced in the quantitative study.

Regarding the broader study, we used a cross-sectional questionnaire, with a non-probabilistic sample and subsequent multivariate statistical analysis. Assisted reproduction clinics in Brazil were the units of analysis, and we aimed to discover their distribution, available services, technologies employed, practices performed, population served and existing forms of funding. We opted to use the REDCap^®^ Program, a web-based application for the construction and management of online surveys and databases. The questionnaire addressed the characterization of services, care and recent changes in practices regarding: the center’s overall time in activity, main characteristic (public or private), the existence of another branch (or lack thereof), number of employees and professionals physically present in the center, available treatments, patient profile, change in light of alterations in statute (CFM, 2013), most frequent reproductive problems seen in the demographic group, the treatment of non-residents in the country, precedence and type of treatment patients sought’’. In order to define the clinics to enter the study, we surveyed their services based on the cross referencing of some databases: medical societies (Brazilian Society for Assisted Reproduction/SBRA), REDLARA (Latin American Network for Assisted Reproduction) and SisEmbrio (National Embryo Production System Registry of the National Agency of Sanitary Surveillance, ANVISA). The Brazilian Society of Assisted Reproduction (SBRA) listed 133 associated centers in its website in 2015, but it is not known which had their own lab. REDLARA encompassed 58 registered clinics in Brazil in 2015 (Zegers-Hochschild *et al*., 2018; 2019). The SisEmbrio registry, established in 2008, only includes clinics that perform ART, adding up to 141 registers in 2015. This procedure was adopted, as there is no data on how many centers are there in the country.

The initial procedure was to contact all the listed clinics by telephone, and requesting the email address to which the form should be sent and the name of the person responsible for providing the service information. Subsequently, the forms were sent in stages, according to regions of the country, to the email addresses informed along with the free and informed consent form. Procedures for resubmission of unanswered forms were adopted up to two times over a 40-day interval.

There were 81 answers between September 2016 and August 2017. There were 2 refusals, 12 questionnaires were accessed with acceptance of the terms of commitment but without being completed, and 81 questionnaires were answered. Among the questionnaires answered, 63.1% represented the southeast region, followed by the south region with 23.8%, the northeast region with 7.1% and the mid-west with 6.0%.

We analyzed 81 questionnaires completed by the clinics, about services in operation in the country, for the years 2016 and 2017. The questionnaires completed and returned corresponded to the clinics actively registered in SisEmbrio and performing ART.

The distribution of the questionnaires answered revealed that the return rate was relatively close to the distribution of the clinics in the country, according to the SisEmbrio registry (2018).

## RESULTS

From the perspective of clinics’ operation time, Table 1 shows that the newest, in operation for up to 10 years, correspond to 23.5%. In this range, three clinics / services indicated to function for up to 2 years; two between 2 and 5 years, and fourteen from 5 to 10 years. The highest percentage of clinics was created in the interval between 11 and 20 years ago (between 1996 and 2005), according to the growth of services reported in the literature. It is also worth noting the significant number of clinics that have operated for more than 20 years, with 32.0%, mostly concentrated in the southeast region.

**Table 1:** Centers according to region, years in operation, number of employees and existence of another unit in Brazil

Region*		Years in operation			Total	Number of employees**						Another unit
		<10	11 - 20	>20		<10	11-20	21-40	41-50	>50	Total	
**NE**	3	3	0	6	3	2	1	0	0	6	1
**MW**	2	2	1	5	0	2	2	0	1	5	1
**SE**	7	24	20	51	13	15	16	4	2	50	13
**S**	7	7	5	19	10	6	3	0	0	19	2
**(%)**	23.5	44.5	32.0	100.0	32.5	31.3	27.5	5.0	3.75	100.0	
**Total**	19	36	26	81	26	25	22	4	3	80†	17

* NE northeast, SE southeast, S south, MW mid-west

** Number of employees include health professionals, administrative and security support.

† One center with no information.

Observing the relationship between the time of a clinics’ operation in relation to the region of the country, in the northeast region we have a uniform distribution in the first two classes (up to 10 years and from 11 to 20 years) with three responses for each. It is the same with the mid-west region, with two responses each and one active for more than 20 years. In the southern region, there is a smaller margin with seven clinics of up to 10 years of operation and seven clinics in the range of 11 to 20 years. In this sense, practically the majority of the regions’ clinics have been in operation for 11 to 20 years. In the case of the southeastern region, there is a higher number of services with a longer time in operation - for more than 20 years - 20 of them. In the northeast there is no clinic registered in operation for more than 20 years among the interviewees.

Considering the size of the establishments, we observed the prevalence among respondents of clinics with small numbers of employees. Of these, 35.4% had up to 10 employees, not only including healthcare professionals, but also administrative support staff. We noticed that 65.9% of the clinics had up to 20 employees. Considering the existing healthcare professionals, there was the following distribution: 100.0% doctors specializing in ART, 88.0% embryologists, 84.5% nurses, 65.5% urologists, 64.2% biologists, 59.5% andrologists, 58.3% psychologists, 8.33% biomedical specialists, 7.14% pharmacists and 4.77% geneticists. Other professionals were mentioned at least once by the centers: anesthesiologists, immunologist, acupuncturists, music therapists, biotechnology engineer and vets.

With reference to the existence of another unit as a parameter to identify the enlargement and/or market concentration, 20.2% of the clinics had another care unit. Of the clinics that declared that they had another unit, 76.5% were in the southeast region, followed by the south region with 11.8%. The location of the other unit, reported by the interviewed clinics, revealed that, in the majority, they were located in another city in the same state of operation as the main clinic with thirteen citations and four of them were located in another state.

Considering the distribution of the clinics according to their characteristics, there was a prevalence of private clinics, with 90.1%. Essentially public services represented 2.5%, corresponding to state or municipal services linked to authorities or healthcare secretaries. Public universities, with 2.5%, corresponded to services linked to universities, which do not charge for procedures and in which users do not need to pay for the medication. Partially public university services represented those in which users needed to pay for medication, with 3.7% of respondents. There is also a private university service, which carries out procedures with low costs for the patients representing 1.2% of the interviewees. Regarding the last four, the treatment coverage is for both low and high complexity cases.

Regarding the existence of connections with another service, 68.0% of respondents declared they performed complementary procedures or techniques with others services. Among the clinics, we had 51.8% with up to 15 employees, followed by those with 16 to 30 employees, with 33.9% of referrals. Concern years of experience, 48.1% of the clinics had been established between 11 and 20 years and 28.0% had been in operation for more than 20 years.

Among the most mentioned procedures performed in collaboration with other services, we have: preimplantation genetic diagnosis as the most mentioned - 43 times; and use of semen banks from 11 responders. The following appears with two references each, vasectomy reversal and egg donation. With one mention, seminal culture and tubal recanalization, mentioned once, each.

According to Table 2, the profile of the population served in the clinics according to the region of the country (allowing simultaneous responses), we have in first place, heterosexual couples with 100.0%; followed by lesbian couples with 77.8%; very closely, there are single women with 74.1%; after this group, the gay couples appear with 39.5%, and single men with 29.6%.

**Table 2:** Profile of population treated according to the region in Brazil

Region*		Heterosexual couples	Female singles	Lesbian couples	Gay couples	Male singles
**NE**	**N**	6	3	5	4	2
**MW**	**N**	5	4	4	2	3
**SE**	**N**	51	42	42	23	15
**S**	**N**	19	11	12	3	4
**Total**	**N**	81	60	63	32	24
	**%**	100.0	74.1	77.8	39.5	29.6

* NE northeast, SE southeast, S south, MW mid-west

Among the main treatments available in assisted reproduction clinics, Table 3 shows that the most reported were intracytoplasmic sperm injection (ICSI) (77/81 clinics), artificial insemination (77/81) and *in vitro* fertilization (73/81). The distribution between the regions does not show significant differences. In its distribution, there is predominance in the southeastern region. In the case of pre-implantation genetic testing (PGT), 84.0% of the clinics claim to engage in collaborative work; otherwise, the majority of clients were being referred to the technique in another center.

**Table 3:** Main treatments available according to the region in Brazil

Region		Total	AI	IVF	ICSI	PGD	Oocyte donation	Embryo donation	Surrogacy
**NE**	**N**	6	6	4	5	5	5	4	3
**MW**	**N**	5	5	5	5	4	4	4	5
**SE**	**N**	51	47	46	48	42	45	38	39
**S**	**N**	19	19	18	19	17	14	13	15
**Total**	**N**	81	77	73	77	68	68	59	62
	**%**	100.0	95.1	90.1	95.1	84.0	84.0	73.0	76.5

A significant number of clinics reported having attended foreigners in 2014 and 2015. That is, 85.1% (n=69) of the clinics reported having seen non-Brazilian residents. Among the most mentioned countries are Angola, the United States of America, Portugal, Italy and Argentina. Among the main treatments sought by foreigners, there are 63 IVF/ICSI; 21 egg donations; 7 PGT, and 6 AI (artificial insemination). The distribution indicates a concentration of these patients in the southeastern region with 63.6%; the south region with 23.6%; the northeast with 7.2%, and the Brazilian mid-west with 5.6%.

Among the reasons given for searching treatment in Brazil, they listed legal issues or restrictions by their countries of origin, 11 respondents; difficulty of access because of treatment costs in the country of origin, 29 respondents. However, the most frequent reason was associated with the quality and effectiveness of the services sought 45 respondents. Other reasons pointed out were some family connection in the city; spouse is Brazilian; there is no service in the country of origin; good references of the doctor and the spoken language.

## DISCUSSION

In the 1990s, studies indicated the existence of 10 private clinics in the country and 7 public and/or university services (Barbosa, 1999; Pereira, 2011). In that context, the specialty started in the country after the first test-tube baby was born in 1984. Today, the SisEmbrio, produced by the National Agency of Sanitary Surveillance (ANVISA, 2018), provides the most accurate record of the number of clinics performing assisted reproduction in Brazil.

However, it registers information only from clinics that perform high complexity procedures, that is, that have banks of cells and germ tissues, with procedures such as *in vitro* fertilization (IVF) and intracytoplasmic sperm injection (ICSI). The registry is produced annually, based on information sent by each clinic in the country, and it is mandatory.

The data obtained from the present study revealed the growth of practices in the country and the diffusion of reproductive techniques. The distribution of the clinics indicates that its expressive concentration continues to occur in the richest region of the country - the southeast. Some other regions have very few clinics, such as the north with five, and there are some states that have none.

Concerning the structure of the centers, we noticed the prevalence of clinics with a small number of employees, opened about 11 years ago. It is worth noting the outstanding technological capacity of these clinics, as only a declining and small number of procedures are not carried out within the premises.

Regarding fund sourcing, there are no relevant changes in the field, since the techniques remain limited to the private initiative, as it has been since the 1990s. In other words, the access of people and couples interested in reproduction depends mainly on the availability of private financial resources (Tavares *et al*., 2016). This implies not only the payment of the procedures performed directly in the clinics - the fertilization cycles - but also the payment of exams and the purchase of medicines. We also noticed that private healthcare insurance plans do not provide coverage for ART procedures, making it clear that the effectiveness of "reproductive rights" is directly related to the socioeconomic status of the interested parties.

On the other hand, public services are, for the most part, university services. According to Souza (2014), given this characteristic, they are dependent on variable resources, depending on the political cycles that influence the determination of the budget amounts allocated to those institutions. That is, the restrictions of public funds in times of economic crisis or even due to the alternation of different political projects in relation to the guarantee and maintenance of those services have an impact on the possibilities of their expansion.

The Unified Healthcare System (SUS) does not foresee coverage of ART procedures among those to be guaranteed within the system, although the Family Planning Act of 1996 (Brazilian Law 9.263) has guaranteed broad reproductive rights, which are not restricted only to access methods of contraception, but also to those of conception.

Even in the scarce public services available, total costs are not always borne by public funding (Souza, 2014). Therefore, the varied classification used in this study sought precisely to confirm the previous field of information: to depend on the service accessed, the user (i) will not make any payment (general public and public university), (ii) will not pay for the treatment, but must buy medicines (partially public university) and, finally, (iii) it will be partially covered by a care institution (private university).

Another dimension of access, availability and financing of ART in the country appears in an initiative of "adequacy" of the prices of the procedures according to family income. This modality represents a significant reduction in the prices practiced, enabling families with smaller incomes to have access to the same resources and procedures enjoyed by those with higher incomes. In these clinics, a "second door of entry" is created for those families with a lower income. In some cases, a "different service" or unit with a different name from the main service is created. The available techniques are the same and although some of these "most popular" units are miles apart, the clinical staff, and especially their “expertise”, are also common to both services. The economic justification of this organization is because, from the fixed costs borne by the clientele with greater purchasing power, it is possible to expand the service for those who could not carry out the procedures without a reduction or discount in prices.

The majority of clinics active in the reproductive field are structured to develop the main techniques in their own facilities, doing little work in collaboration or referral to other complementary techniques or procedures. In this context, some of our interviewees revealed performing procedures and diagnostic support in other services. The most commonly cited technique is PGT (Preimplantation genetic testing), a highly specialized genetic test that involves a range of techniques used to evaluate the genetic condition of the embryo obtained by IVF or ICSI.

As for the techniques available, the ICSI technique was developed to respond to male infertility problems, but in the perspective of a practice that occurs in several other fields of technology, it ended up being configured as the most used technique, regardless of the problem being treated being relative to the male factor. This trend can also be observed in several other countries (Tannus *et al*., 2017). *In vitro* fertilization itself, when emerged as a technique, was also gradually supplanting the use of existing ones.

The access of new subjects to reproductive technologies is contributing to the expansion of the reproductive market. Our study indicates that the search for homoparentality (lesbian or gay) and monoparentality has been increasing. This perspective can be perceived by parts of the questionnaire carried out in the study, as well as by the increased demand for obtaining genetic material from third parties, observable from the publication, by ANVISA, of the 1^st^ and 2^nd^ Report of Seed Samples (*Relatório de Amostras Seminais*) for use in Assisted Human Reproduction (ANVISA, 2017; 2018). Among the interviewees, there was a significant mention of the 2121 Resolution (CFM, 2015) which led to an increase in the number of lesbian and gay couples in treatment.

In addition, the study indicates that the changes produced in the medical regulations, which regulate the field of assisted reproduction, have increased the population served by services, the greater presence of lesbian couples, female singles, gay couples and, to a lesser extent, male singles. What seems to drive modifications in resolutions are the "demands" or "pressures" of society itself, that is, couples or individuals seeking assisted reproduction practices and clinics. This is because, often, these demands point to the dimensions or possibilities of ART use, which confront, or have not yet been incorporated, by the current resolution.

The possibility that homosexual couples and singles have access to ART, although they do not present problems of infertility, is an example of how the resolutions end up ratifying changes caused by pressure from society and that these were interpreted differently by the specialists until their incorporation into the regulatory document. This is not another expression of this fact that in the CFM resolutions, from 2010, "infertility problems" would be replaced by "assisting in human reproduction" and, likewise, that from 2013, the possible use of ART by homosexual couples and single people would be explicitly included in the text. In fact, what results in the text of the resolution depends in varying degrees on the interactions between doctors and patients, demonstrating that the technologies developed from the identification of certain diseases are being re-signified by the subjects' conceptions about family, paternity and maternity. It is these conceptions that reconfigure the understanding of the limits and potentialities of the techniques available, and the diseases that are at their origin.

The most widely recognized characteristic in the literature about the topic (Brown & Webster, 2004; Waldby & Mitchell, 2006; Webster, 2007; Cooper & Waldby, 2014), the increase of access, or the diffusion of techniques previously restricted to a specific public, reproduces, also in biomedical technologies, what appears to be the pattern of technological development in contemporary societies. That is, the gradual incorporation of those that can be achieved by a set of instruments and procedures initially circumscribed to well-defined cases.

However, if part of the modifications of the resolutions can be credited to the pressures, conceptions and desires of those who "still have not been reached by the technique", the other part refers to the alterations occurred in the very development of technologies in this case, especially in the biomedical technologies sector. The emergence of new instruments, new techniques, new drugs and new procedures represents a determinant of the increase in ART success rates or effectiveness. Thus, as the introduction of innovations produces alterations in the results of clinical practice, there is scope for the constitution of some certainties, even of temporary duration, regarding the age limits for access, for donation, for the possibilities of using own genetic material and of third parties (cryopreservation), and the effectiveness and efficiency of one procedure when compared to another. It is these "provisional certainties" that conform the resolutions and impose the periodic necessity for their revisions according to the specialists in the field.

Relevant data refers to the participation of foreign patients being treated in Brazil. A significant number of clinics reported services to non-residents in the country in 2014 and 2015. Among the most mentioned countries were Angola, the United States of America, Portugal, Italy and Argentina. In the case of couples from Angola, despite the massive global expansion of ART services over the past years, they remain inaccessible in Sub-Saharan Africa. Inhorn & Patrizio (2015) note that seven out of the fifteen nations have a single IVF (in vitro fertilization) clinic. The exception in this context are Ghana, Nigeria and South Africa that seem to have better-structured services. This picture of ART shortages contrasts with the high estimate of infertility in the region (Machin *et al*., 2018).

This flow of patients from other countries is associated with different regulations among countries, which may involve prohibition of access to some groups of users, existence of restrictive criteria regarding coverage of practices in national healthcare systems, higher costs involving treatments and prohibition of some practices (Gürtin & Inhorn, 2011). This phenomenon, linked to the expansion of markets in a globalized context, is also driven by the possibilities opened by digital connection. Displacements may be indicated by medical professionals in their country of residence, by indication of people who have already had treatment, by consulting agencies specialized in this practice or by direct search on the internet.

Although the rules in Brazil do not configure liberated practices such as those involving the purchase and sale of genetic material from third parties or surrogacy, clinics located in the country have demonstrated potential to appear be a destination for patients seeking reproductive treatment.

## CONCLUSION

Since the introduction of assisted reproduction technologies in Brazil (1984) to the present day, there has been a significant expansion of offers in this country, although with uneven distribution across the territory, the southeast region having the largest number of clinics. 

Inequality in the field of ART also manifests itself in access, markedly dependent on its own financial resources and in the small number of public-assisted reproductive healthcare services, making clear the influence of the financial aspect in seeking these services.

The CFM resolutions on the subject were gradually increasing the access of subjects to ART, regardless of the existence of a health issue that justified its use, as it was previously. With this, new family configurations and new subjects of rights were affected by the modifications made, changing the prevailing conception of "health care" to "assistance to new family configurations". Because of this expansion and despite the financial restrictions derived from the prevalence of private services in the area, the search for assisted reproduction has expanded in the country. The growth of ART services in Brazil is also reflected in the increase and the significant demand of foreigners for services here.

## References

[r1] Barbosa RM (1999). Desejo de filhos e infertilidade: um estudo sobre a reprodução assistida no Brasil.

[r2] Brazil, ANVISA Agência Nacional de Vigilância Sanitária (National Health Surveillance Agency) (2017). 1° Relatório de Importação de Amostras Seminais para uso em Reprodução Humana Assistida.

[r3] Brazil, ANVISA Agência Nacional de Vigilância Sanitária (National Health Surveillance Agency) (2018). 2º Relatório de dados de importação de células e tecidos germinativos para uso em reprodução humana assistida.

[r4] Brazil, ANVISA Agência Nacional de Vigilância Sanitária (National Health Surveillance Agency) (2016). 9º Relatório do Sistema Nacional de Produção de Embriões. SisEmbrio.

[r5] Brazil, CFM - Conselho Federal de Medicina (Federal Council of Medicine) (2010). Resolução 1957/2010.

[r6] Brazil, CFM - Conselho Federal de Medicina (Federal Council of Medicine) (2013). Resolução 2013/2013.

[r7] Brazil, CFM - Conselho Federal de Medicina (Federal Council of Medicine) (2015). Resolução 2121/2015.

[r8] Brazil, CFM - Conselho Federal de Medicina (Federal Council of Medicine) (2017). Resolução 2168/2017.

[r9] Brown N, Webster A (2004). New medical technologies and society: reordering life.

[r10] Cooper M, Waldby C (2014). Clinical Labor. Tissue Donors and Research Subjects in the Global Bioeconomy.

[r11] Gürtin Z, Inhorn MC (2011). Introduction: travelling for conception and the global assisted reproduction market. Reprod Biomed Online.

[r12] Inhorn MC, Patrizio P (2015). Infertility around the globe: new thinking on gender, reproductive technologies and global movements in the 21st century. Hum Reprod Update.

[r13] Machin R, Augusto MHO, Mendosa D (2018). Cross-border reproduction: the reproductive market in Angola and Brazil. Papeles CEIC. Int J Collect Identity Res.

[r14] Pereira DHM (2011). A história da reprodução humana no Brasil. FEMINA.

[r15] Souza MCB (2014). Latin American and access to assisted reproductive techniques: a Brazilian perspective. JBRA Assist Reprod.

[r16] Tannus S, Son WY, Gilman A, Younes G, Shavit T, Dahan MH (2017). The role of intracytoplasmic sperm injection in non-male factor infertility in advanced maternal age. Hum Reprod.

[r17] Tavares R, Cunha G, Aguiar L, Duarte SC, Cardinot N, Bastos E, Coelho F (2016). Socioeconomic profile of couples seeking the public healthcare system (SUS) for infertility treatment. JBRA Assist Reprod.

[r18] Waldby C, Mitchell R (2006). Tissue Economies. Blood, organs and cell lines in late capitalism.

[r19] Webster A (2007). Health, Technology and Society. A Sociological Critique.

[r20] Zegers-Hochschild F, Schwarze JE, Crosby J, Musri C, Urbina MT, Latin American Network of Assisted Reproduction (REDLARA) (2018). Assisted reproductive techniques in Latin America: the Latin American Registry, 2015. Reprod Biomed Online.

[r21] Zegers-Hochschild F, Schwarze JE, Crosby J, Musri C, Urbina MT (2019). Assisted Reproductive Techniques in Latin America: The Latin American Registry, 2015. JBRA Assist Reprod.

